# Cyclodextrin-Based Hybrid Polymeric Complex to Overcome Dual Drug Resistance Mechanisms for Cancer Therapy

**DOI:** 10.3390/polym13081254

**Published:** 2021-04-13

**Authors:** Lingjie Ke, Zhiguo Li, Xiaoshan Fan, Xian Jun Loh, Hongwei Cheng, Yun-long Wu, Zibiao Li

**Affiliations:** 1 School of Pharmaceutical Science, Xiamen University, Xiamen 361102, China; 32320191153429@stu.xmu.edu.cn (L.K.); lizhiguo@stu.xmu.edu.cn (Z.L.); 2 State Key Laboratory for Modification of Chemical Fibers and Polymer Materials, Donghua University, Shanghai 201620, China; xsfan@dhu.edu.cn; 3 Institute of Materials Research and Engineering (IMRE), Agency for Science, Technology and Research (A*STAR), 2 Fusionopolis Way, #08-03 Innovis, Singapore 138634, Singapore; lohxj@imre.a-star.edu.sg

**Keywords:** drug resistance, gene delivery, polymeric complex, cancer therapy

## Abstract

Drug resistance always reduces the efficacy of chemotherapy, and the classical mechanisms of drug resistance include drug pump efflux and anti-apoptosis mediators-mediated non-pump resistance. In addition, the amphiphilic polymeric micelles with good biocompatibility and high stability have been proven to deliver the drug molecules inside the cavity into the cell membrane regardless of the efflux of the cell membrane pump. We designed a cyclodextrin (CD)-based polymeric complex to deliver chemotherapeutic doxorubicin (DOX) and Nur77ΔDBD gene for combating pumps and non-pump resistance simultaneously. The natural cavity structure of the polymeric complex, which was comprised with β-cyclodextrin-graft-(poly(ε-caprolactone)-adamantly (β-CD-PCL-AD) and β-cyclodextrin-graft-(poly(ε-caprolactone)-poly(2-(dimethylamino) ethyl methacrylate) (β-CD-PCL-PDMAEMA), can achieve the efficient drug loading and delivery to overcome pump drug resistance. The excellent Nur77ΔDBD gene delivery can reverse Bcl-2 from the tumor protector to killer for inhibiting non-pump resistance. The presence of terminal adamantyl (AD) could insert into the cavity of β-CD-PCL-PDMAEMA via host-guest interaction, and the releasing rate of polymeric inclusion complex was higher than that of the individual β-CD-PCL-PDMAEMA. The polymeric inclusion complex can efficiently deliver the Nur77ΔDBD gene than polyethylenimine (PEI-25k), which is a golden standard for nonviral vector gene delivery. The higher transfection efficacy, rapid DOX cellular uptake, and significant synergetic tumor cell viability inhibition were achieved in a pump and non-pump drug resistance cell model. The combined strategy with dual drug resistance mechanisms holds great potential to combat drug-resistant cancer.

## 1. Introduction

Despite the ongoing efforts to develop novel anticancer drugs to fight various types of cancers, drug resistance still leads to a high mortality rate of patients [1,2,3,4]. So far, multidrug resistance (MDR) and low drug solubility are the main reasons that limit the efficacy of most chemotherapeutic drugs [5,6]. Unlike active drug resistance, MDR could induce cancer cells to become cross-resistant to both active drugs and other chemotherapeutic drugs, which leads to the failure of chemotherapy and lead to further development of disease status [7,8].

Doxorubicin (DOX, also named as adriamycin), a typical anthracycline chemotherapeutic drug, has been widely used in the treatment of leukemia, liver cancer, and malignant lymphoma. However, many side effects and frequent MDR development limit its clinical application [9,10,11]. Drug efflux mediated by ATP-binding cassette (ABC) drug transporters is an essential reason of MDR in many chemotherapeutic drugs [12,13,14]. These drug transporters are generally overexpressed on the membrane surface of drug-resistant cancer cells. Typical efflux transporters include P-glycoprotein (P-gp), breast cancer resistance protein (BCRP), and multidrug resistance-associated protein 1 (MRP1) [15]. Among the above, multidrug resistance mediated by P-gp, also known as multidrug-resistant protein 1 (MDR1) encoded by ABC subfamily member 1 (ABCB1), is the most common in clinical therapy of liver, kidney, and colon cancer, which endows cancer cells an ability to excrete exogenous chemotherapeutic drugs (i.e., doxorubicin) [16]. Besides, there is no effective targeted drug for MDR1 that has been developed so far [17,18]. In addition to a pump efflux resistance mechanism demonstrated by protein transporters, non-pump resistance (i.e., some anti-apoptosis mediators) is another important cause of MDR [19]. Many studies have shown that Bcl-2 protein is overexpressed in many malignant drug-resistant tumor cells, as the protector of tumor cells against chemotherapy and the overexpression of Bcl-2 confer the pro-oncogenic function through apoptosis inhibition [20,21]. Bcl-2 expression is mainly located on the mitochondrial outer membrane, with an N-terminal transmembrane region controlling mitochondrial membrane voltage, which leads to inhibition of tumor cell apoptosis by inhibiting mitochondrial cytokines (i.e., cytochrome C and apoptotic factors) release [22,23,24]. Orphan nuclear receptor Nur77 (also named as TR3/NGFI-B) has been proven to be located between the BH3 and BH4 domains of mitochondrial Bcl-2 protein to change the conformation of the Bcl-2 protein family, reversing the anti-apoptotic mechanism into a pro-apoptotic one, which might be a solution to overcome non-pump resistance [25,26].

In recent years, the intelligent and controllable nanomedicine caused a lot of attention in order to adapt to the complex environment in the human body and many different surface features. The shape of hydrophobic nano drugs have been developed through cell endocytosis into the cells, even in intracellular transport, which have great potential as a drug delivery vector through active/passive targeting of osmosis [27]. Based on this, a variety of biomaterials with good intracellular endocytosis have been developed to reverse MDR, including liposomes, polymer micelles, etc. [28]. Compared with liposomes with water instability and poor ability to carry fat soluble drugs, amphipathic polymeric micelles with good biocompatibility, drug loading capacity, and water stability can encapsulate chemotherapeutic drugs by physical affinity or chemical bonding and deliver inside the cell membranes, thus, escaping the cell membrane pump efflux [12,29,30,31]. Cyclodextrin (CD)-based materials easily form a natural loading region due to the existence of a core cavity, in which the dendritic polymer structure with cyclodextrin as the core is easy to be modified and allows element assembly, which is easy to occur as a host-guest interaction with drugs, achieving efficient drug delivery [32,33,34].

To overcome the limitations of the pump and non-pump resistance of doxorubicin simultaneously, we designed a polymeric inclusion complex with rapid cellular uptake to overcome the drug pump efflux. The therapeutic Nur77ΔDBD gene was co-delivered to reverse the Bcl-2 mediated non-pump drug resistance. In this work, β-CD-PCL-AD and β-CD-PCL-PDMAEMA copolymers were formed by grafting adamantyl (AD) and positively charged PDMAEMA on the end of hydrophobic fragment polycaprolactone (PCL), with β-CD-PCL as the basic unit. The cavity structure of β-CD was suitable for drug loading, and the complexation of the AD terminal and CD made the terminal AD of β-CD-PCL-AD have the potential to be inserted into the CD cavity of β-CD-PCL-PDMAEMA to form an inclusion complex. Such a spatial structure constructed by a physical interaction contributed to the increase of drug loading. This polymeric inclusion complex is expected to overcome multiple drug resistance of DOX and provide an effective strategy for tumor chemotherapy.

## 2. Materials and Methods

### 2.1. Materials

Penicillin-Streptomycin (10,000 U/mL) antibiotics (Gibco, 15140122), Bicinchoninic acid (BCA) Protein Assay Kit (Pierce, 23227), Dulbecco’s Modified Eagle Medium (DMEM) (Gibco, 11965092), Fetal Bovine Serum (FBS) (Gibco, 10270106), and Opti-MEM^TM^ Reduced Serum Medium (Gibco, 31985062) were purchased from ThermoFisher Scientific (Waltham, MA, USA). Hexadimethrine bromide (polybrene, 107689), puromycin dihydrochloride (>98%, P9620), 4′,6′-diamidino-2-phenylindole (DAPI, 10236276001), and doxorubicin were purchased from Sigma-Aldrich (St. Louis, MI, USA). Nucleic Acid Gel Stain (YeaRed, 10202ES76), Agarose (10208ES76), Cell Counting (CCK-8) Kit (40203ES76), total RNA extraction reagent (Trizol, 10606ES60), first Strand cDNA Synthesis Kit (11121ES60), and Hieff™ qPCR SYBR^®^ Green Master Mix (11203ES08) were purchased from Yeasen Biotech (Shanghai, China). Tris base-acetic acid- EDTA (TAE) buffer (ST716), DNA loading (D0071), and Relilla luciferase reporter kit (RG017) were acquired from the Beyotime company (Shanghai, China).

### 2.2. The Preparation of the Inclusion Complex

The synthesis methods of the β-CD-PCL*-b-*PDMAEMA polymer are as described in our previous article [2]. ^1^H NMR spectra of β-CD-PCL-PDMAEMA was detected by nuclear magnetic resonance (NMR, JEOL JNM-ECA 500 MHz, JEOL, Akishima, Japan) at room temperature and CDCl_3_ as solvent, as shown in Supplementary Figure S1. In addition, the following protocol was employed to self-assemble β-CD-PCL-AD/CD-PCL*-b-*PDMAEMA for the inclusion complex. β-CD-PCL-AD and CD-PCL*-b-*PDMAEMA were synthesized as our previous reports [2,33]. Both were dissolved in 20% tetrahydrofuran (THF) aqueous (5 mL) solution, respectively, and the molar ratio of β-CD-PCL-AD and β-CD-PCL*-b-*PDMAEMA was 1:2. Then, the solution of β-CD-PCL*-b-*PDMAEMA was added into the β-CD-PCL-AD solution at a rate of 2 mL/h under violent stirring at room temperature for 48 h. The inclusion complex was obtained by filtering and freeze drying.

### 2.3. The Gel Retardation Assay

The binding efficiency of the inclusion copolymer with plasmid DNA (pDNA) was assessed by a gel retardation assay, as follows below. Briefly, the pDNA (200 ng) was diluted in deionized water (20 µL) and added an β-CD-PCL-AD/β-CD-PCL*-b-*PDMAEMA inclusion complex at various N/P ratios from 1:5 to 4:1. The two solutions were mixed gently and incubated for 30 min at room temperature. In addition, then DNA loading buffer was added to each sample. The electrophoresis was utilized with 1.5% agarose gel in TAE buffer at a voltage of 100 V for 30 min. After that, the agarose gel was scanned by using an ultraviolet (UV) image system (Bio-Rad, Hercules, CA, USA).

### 2.4. Dynamic Light Scattering (DLS) Assay

The particle sizes and zeta potentials of β-CD-PCL-AD/β-CD-PCL*-b-*PDMAEMA inclusion complex with enclosing plasmid DNA (pDNA) were measured using Malvern Zetasizer equipment at 25 °C. The data of the particle size and zeta potential were collected with detection angles of scattered light at 90° and 15°, respectively, and the dispersant was set as phosphate-buffered saline (PBS) buffer.

### 2.5. Cell Culture

The normal human embryonic kidney HEK 293T cells and liver cancer HepG2 cells were obtained from American Type Culture Collection (ATCC). Both the two cell lines were maintained in Dulbecco’s Modified Eagle’s Medium (DMEM), supplemented with 10% fetal bovine serum (FBS), 100 U/mL penicillin, and 100 µg/mL streptomycin. In addition, due to the enhanced cell membrane permeability in the transfection process, the culture medium was required to be free of penicillin and streptomycin for preventing antibiotics cytotoxicity against cells and reducing the error of transfection efficiency. All cell lines were cultured in a humidified 37 °C and 5% CO_2_ incubator.

### 2.6. Induction of a DOX Drug-Resistant Cell Line

DOX drug-resistant HepG2/ADR cells were induced by using a continuous and increasing concentration of DOX treatment. Briefly, the HepG2 cells were seeded in a 6-cm cell culture plate, and treated with DOX with an initial concentration at 0.1 µmol/L. During the induction, the culture medium was supplemented with a reduced serum level (about 5% FBS). The dead cells were removed and further treated with DOX in a dose gradient manner. After two months of induction, the cells were stably cultivated in the culture medium containing 0.5 µmol/L DOX. The resistance index was assessed to evaluate the drug-resistant capacity, and the resistance index (RI) was measured by the ratio of the IC50 of drug-resistant induced cells between the parental cells.

### 2.7. Cell Viability Measurement

The HEK293T cells and HepG2/MDR1-Bcl2 cells were seeded in 96-well plates at the density of 7 × 10^3^ cells/well and 5 × 10^3^ cells/well, respectively. In addition, when further incubated for 24 h, the cells were treated with inclusion polymer and PEI-25k (25,000 Da) for 24 h at various concentrations. The relative cell viability was evaluated by the cell counting kit (CCK-8) according to the manufacture of the product’s protocol. Briefly, 10 µL/well CCK-8 reagents (10% medium volume) were added to the plate, and further cultured for 3 h in the incubator. The absorbance value was assessed by a microplate reader with a wavelength at 450 nm. Combination index (CI) of DOX and Nur77ΔDBD for inclusion complex/Nurr77ΔDBD-DOX across the fraction affected (*f*_a_) was calculated based on Chou Talalay’s isobolographic method by using software CompuSyn. CI < 1, CI = 1, and CI > 1 indicate synergism, additive effect, and antagonism, respectively [31,35].

### 2.8. Real-Time PCR (RT-PCR) Analysis

Total RNA of the cells was extracted by using the total RNA extraction reagent, and then the RNA was reversely transcribed into cDNA using the first-strand cDNA synthesis kit with a genomic DNA (gDNA) eraser. After that, PCR amplification was evaluated by using a SYBR-Green PCR kit in ABI 7500 thermocycler, and the settlement of the procedure was referred to the protocol of the product. All primers were synthesized by the Sangon company, and the specific primer sequence for each targeting gene were listed as follows: for human MDR1, forward sequence: 5′-CGA CAG GAG ATA GGC TGG TT-3′, reverse sequence: 5′-AGA ACA GGA CTG ATG GCC AA-3′; for human Bcl2, forward sequence: 5′-TTC TTT GAG TTC GGT GGG GT-3′, reverse sequence: 5′-CTT CAG AGA CAG CCA GGA GA-3′; for human GAPDH, forward sequence: 5′-AGG TCG GAG TCA ACG GAT TT-3′, reverse sequence: 5′-ATC TCG CTC CTG GAA GAT GG-3′; The relative gene expression levels were analyzed by using the 2^-^^ΔΔCT^ method. GAPDH (Glyceraldehyde-3-Phosphate Dehydrogenase) was utilized to normalize the target gene expression levels. All experiments were performed in triplicate.

### 2.9. Construction of HepG2/MDR1-Bcl2 Cells

HepG2 cells with overexpression of MDR1 and Bcl2 genes were generated by using the lentiviral infection system. The detailed procedure is as follows. Briefly, the plasmid of pCDH-MDR1, pCDH-Bcl2, and a control vector were transfected into HEK293T cells with the assistant pCMV-VSV-G and pCMV-dR8.91 vector, and the lentivirus particles were collected for infection in HepG2 cells. After 72 h of the infection, the infected cells were treated with 5 µg/mL puromycin for selecting the stably positive expressing cells. A real-time polymerase chain reaction (PCR) was conducted to verify the availability of the stably overexpressing cells. To maintain the availability of the expressing cells, HepG2/MDR1-Bcl2 cells with double overexpressing MDR1 and Bcl2 genes were maintained in complete medium with 2 μg/mL of puromycin added.

### 2.10. Gene Transfection Efficiency Evaluation

The HEK293T cells and HepG2 cells with overexpressing MDR1 and Bcl2 were seeded in 24-well plates and further cultured. When the ratio of cell confluent was about 60%–70%, the cells were subjected to an in vitro luciferase reporter assay by using the Renilla luciferase system for gene transfection efficiency evaluation, as follows. Briefly, the cell medium was exchanged with the Opti-medium, and the polymer/gene complex was prepared as previously described. After being incubated for 24 h, the cells were washed gently with cold PBS buffer twice and lysed in reporter lysis buffer for 30 min on ice. The cell lysis was collected to 96-well white CulturPlate^TM^ (PerkinElmer), and then 100 µL/well Renilla luciferase reaction reagent was added to plates quickly. The value was measured by using a multimode plate reader at 480 nm. The luciferase reporter activity was expressed in the relative light unit (RLU) per milligram of protein in cell lysis (RLU/mg protein). The protein weight of the per well in lysis was measured by the BCA protein assay, according to the manual.

### 2.11. Fluorescence Microscopy Analysis

The HepG2/MDR1-Bcl2 cells were transfected EGFP plasmid with β-CD-PCL-AD/β-CD-PCL*-b-*PDMAEMA inclusion complex, and the N/P ratio was 4. PEI-25k was used as a control for comparison and the N/P ratio was 8. The procedure of preparation of the copolymer/pDNA transfection system was described before. After incubation for 24 h, the cells were washed with phosphate-buffered saline (PBS) buffer to remove the floating cell debris. The fluorescence cells were photographed by Zeiss fluorescent microscopy.

### 2.12. Confocal Microscopy Assay

The DOX cellular uptake experiment was evaluated by confocal fluorescence microscopy. The HepG2/MDR1-Bcl2 cells were seeded at 5 × 10^3^ cells/well in a 12-well plate with glass coverslips. After being cultured for 24 h, the cells were treated with a β-CD-PCL-AD/β-CD-PCL*-b-*PDMAEMA/DOX@NPs complex or DOX only for 12 h. After that, the cells were washed with cold PBS buffer twice and fixed with 4% paraformaldehyde for 10 min at room temperature. 4′,6′-diamidino-2-phenylindole (DAPI) was used for staining the nucleus of cells. To avoid fluorescence quenching, the operation should be away from light.

### 2.13. Statistical Analysis

Data was expressed as a mean standard deviation (SD). The significant difference of the data was determined by using a student’s *t*-test between the two groups. * *p* < 0.05 was considered as statistical significance.

## 3. Results and Discussion

### 3.1. Gene Binding and Drug Loading Ability of the Polymer Inclusion Complex

**Scheme 1:**
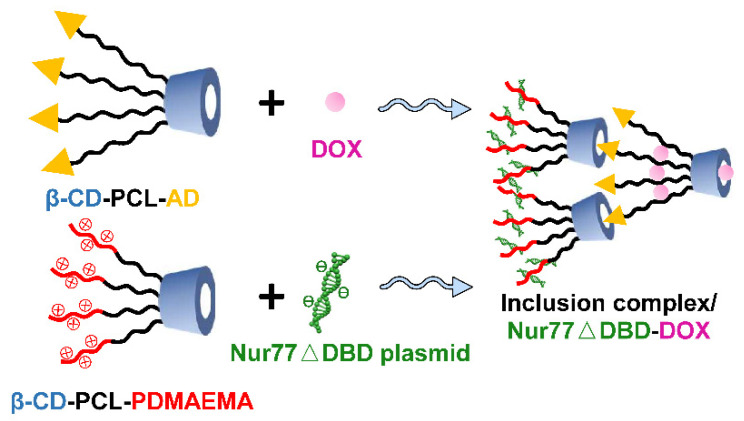
Schematic representation of the host-guest self-assembly process of inclusion complex/Nur77ΔDBD-DOX.

**Figure 1:**
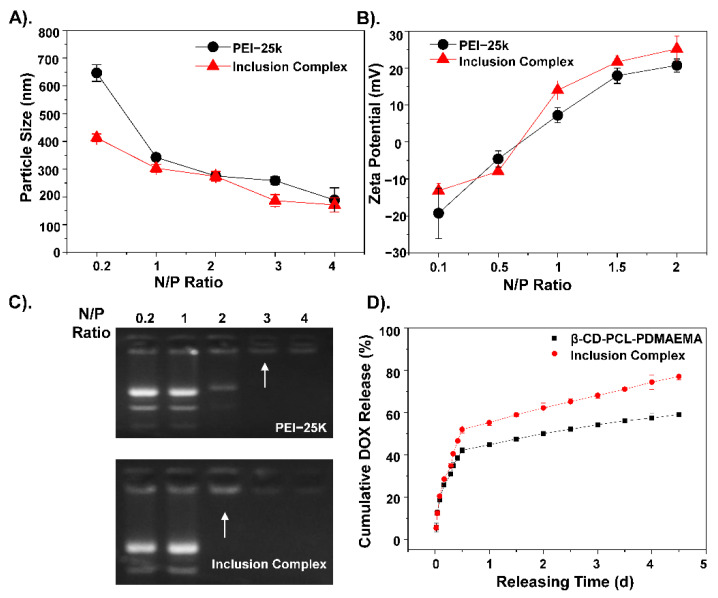
The corresponding hydrate (**A**) particle size and (**B**) zeta potential of inclusion complex/Nur77ΔDBD nanocomposites with a gradient N/P ratio. (**C**) Gel retention diagram of PEI/Nur77ΔDBD and inclusion complex/Nur77ΔDBD complexes under a gradient N/P ratio. (**D**) Cumulative Doxorubicin (DOX) release curves of β-CD-PCL-PDMAEMA alone and inclusion complex.

The inclusion complex was cross-linked together mainly through two forms of self-assembly of β-CD-PCL-based polymers, and the assembly process, as shown in Scheme 1. By detecting the concentration of unloaded drugs, it was found that the loading rate of this inclusion complex was high to 86% compared to the 65% loading rate of β-CD-PCL-AD alone. The presence of AD helped the β-CD-PCL-AD polymer to insert into the cyclodextrin cavity of β-CD-PCL-PDMAEMA, greatly increasing the possibility of cross-linking and the space for drug loading. In order to compare the gene binding rate of the inclusion complex, the commonly used PEI-25K was used as the control group. Under different N/P ratios, the binding effect of polymers to plasmid DNA (pDNA) is different. Therefore, the particle size and potential of the final polymer are different. Compared with PEI-25k, the inclusion complex has smaller and more stable particle sizes (Figure 1A). In addition, the high positivity of the inclusion complex indicates that it has better gene binding ability than PEI-25k (Figure 1B), which is also shown in the gel retardation assay with a lower N/P ratio (Figure 1C). As the result of drug accumulative release in Figure 1D shows, the final DOX release amount of the inclusion complex is more than that of β-CD-PCL*-b-*PDMAEMA, reaching nearly 80%, which indicates that the inclusion complex has a better accumulative DOX release effect than β-CD-PCL*-b-*PDMAEMA. This is attributed to the insertion of terminal AD in β-CD-PCL-AD into CD in β-CD-PCL*-b-*PDMAEMA, forming a special inclusion complex structure with many CD cores. Each CD releases drugs at the same time, which causes the initial release rate of drugs to increase, and the final accumulation amount to also increase. Therefore, this stable inclusion complex is expected to be a suitable gene and drug delivery vector.

### 3.2. The MDR of DOX Is Related to MDR1 and Bcl-2

**Figure 2:**
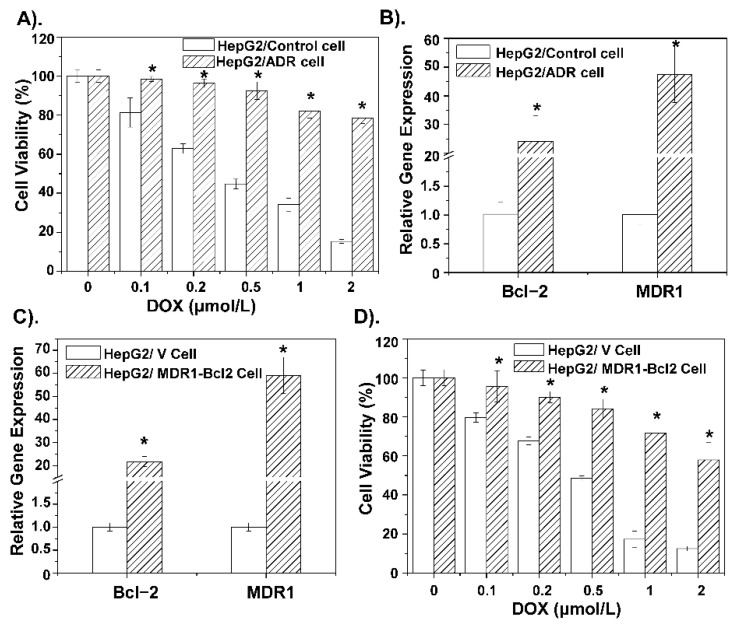
(**A**) Cell viability inhibition analysis of DOX alone incubated with HepG2 cells and HepG2 drug-resistant cells. (**B**) Expression levels of Bcl-2 and MDR1-related genes in HepG2 cells and HepG2 drug-resistant cells. (**C**) Relative expression levels of Bcl-2 and MDR1 genes in self-constructed HepG2/MDR1-Bcl2 cells compared with primary HepG2 cells. (**D**) Cell viability inhibition analysis of gradient concentration DOX incubated with HepG2 cells and HepG2/MDR1-Bcl2 cells. * *p* < 0.05 was considered as statistical significance.

To further understand details about the DOX-related pump and non-pump resistance, this study conducted genetic analysis based on a public expression profiling array GSE125180 (Figure S2), which is a dataset of expression data from doxorubicin-resistant hepatoma cells. The results showed DOX resistant-related genes were enriched in the membrane by cellular component (CC) analysis, and the molecular function (MF) analysis showed these differential genes were enriched in gated channel activity, which play important roles in pump and non-pump drug resistance [36,37]. Moreover, MDR1 is located in the cellular membrane, and Bcl-2 is mainly located in the mitochondrial membrane and functions as a gated channel. Furthermore, the overexpression of MDR1 and Bcl-2 in different cancer types were shown in supporting Figure S3. Therefore, construction of MDR1 and the Bcl-2 overexpressing cell line could obtain further understanding about the DOX-related pump and non-pump resistance. To further verify the role of MDR1 and Bcl2 in the mechanism of DOX resistance, HepG2/ADR cells and HepG2/MDR1-Bcl2 cells were constructed. As shown in Figure 2A, the HepG2/ADR cell has clear DOX resistance relative to the HepG2/Control cell, which demonstrates the successful construction of DOX-resistant cells. Subsequently, RT-PCR results show that the relative gene expression levels of Bcl-2 and MDR1 in DOX-resistant cells are significantly increased (Figure 2B). Then, HepG2/MDR1-Bcl2 cells were used to conduct the corresponding RT-PCR detection, and the results show that the relative expression levels of MDR1 and Bcl-2 genes in HepG2/MDR1-Bcl2 cells were significantly increased, and the related expression levels were similar to those in DOX-resistant cells (Figure 2C). The drug resistance of the HepG2/MDR1-Bcl2 cell to DOX was also demonstrated in the cell viability inhibition analysis in Figure 2D. This suggests that the multi-resistance of HepG2 cells to DOX is not only related to the pump resistance factor, such as the protein transporter MDR1, but also the non-pump resistance factor, such as the tumor cell protectant Bcl-2 protein.

### 3.3. Transfection Ability Evaluation of Inclusion Complex in MDR Cells

**Figure 3:**
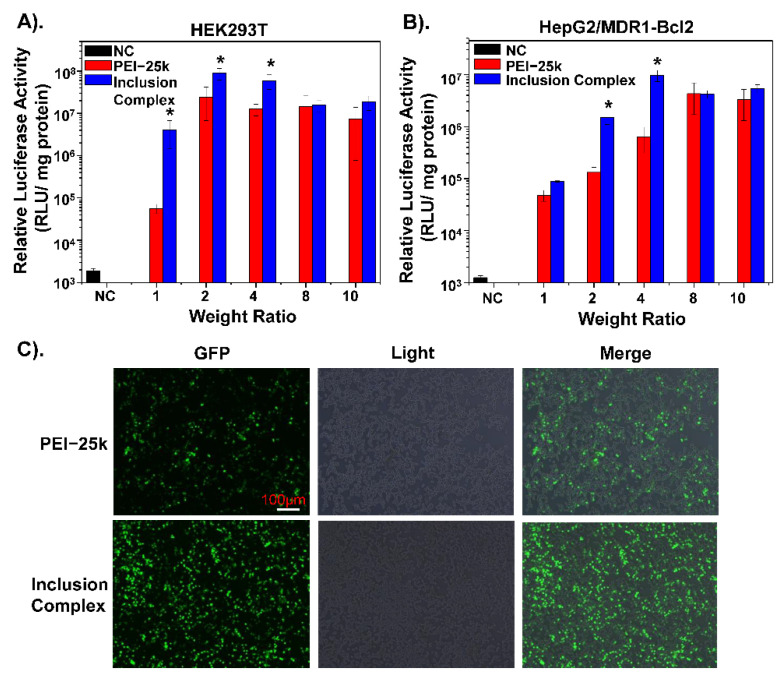
The Luciferase expression efficiency of PEI/Nur77ΔDBD, inclusion complex/Nur77ΔDBD complexes (gradient weight ratios, from 1 to 10) in (**A**) HEK293T cells, and (**B**) HepG2/MDR1-Bcl2 cells. (**C**) Fluorescence expression of EGFP of HepG2/MDR1-Bcl2 cells co-incubated with inclusion complex/Nur77ΔDBD complex with an N/P ratio of 4 for 24 h compared with PEI/Nur77ΔDBD with an N/P ratio of 8. * *p* < 0.05 was considered as statistical significance.

To explore the transfection ability of the inclusion complex, a luciferase reporter assay was conducted. As shown in Figure S4, the transfection abilities of three different experimental treatments under different N/P ratios were evaluated in HepG2 cells. The result shows that, when the N/P ratio is below 8, the transfection effect-containing complex is the best. However, when the N/P ratio exceeds 8, the transfection ability of the inclusion complex is weakened due to the high positivity of the polymer. Besides, in HEK293T cells commonly used for transfection, the transfection ability of contents was better than that of PEI-25k. The same result is also shown in HepG2/MDR1-Bcl2 drug-resistant cells (Figure 3A,B). This indicates that the inclusion complex can effectively deliver the Nur77ΔDBD gene into drug-resistant cells and fully express it. Besides, the HepG2/MDR1-Bcl2 cells were transfected with an EGFP plasmid with an inclusion complex under an N/P ratio of 4 and with PEI-25k under an N/P ratio of 8. As illustrated in Figure 3C, fluorescence microscopy analysis demonstrates that the expression of green fluorescent protein in the polymeric inclusion complex group was significantly higher than that in the PEI-25k group, which proves that the inclusion complex is a better vector for transfection of the Nur77ΔDBD gene into HepG2/MDR1-Bcl2 cells. The inclusion complex is highly electrically positive and has a smaller particle size, allowing it to bind and deliver genes into cells efficiently.

### 3.4. Inclusion Complex Effectively Deliver DOX and Nur77ΔDBD to Overcome MDR

**Figure 4:**
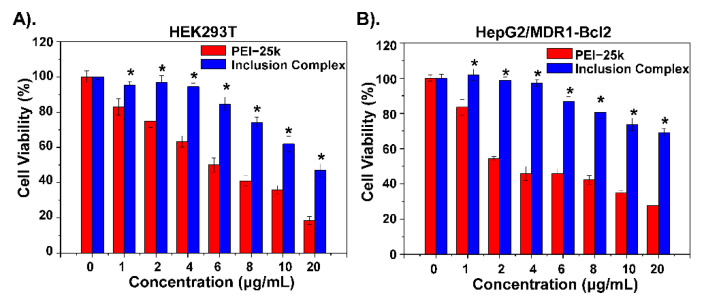
Cell viability inhibition analysis of inclusion complex on (**A**) HEK293T cells and (**B**) HepG2/MDR1-Bcl2 cells compared to PEI-25k alone. * *p* < 0.05 was considered as statistical significance.

**Figure 5:**
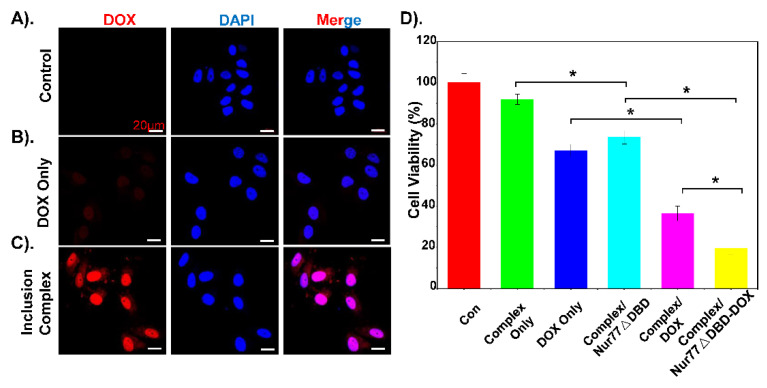
Cell uptake images of (**A**) PBS, (**B**) free DOX, and (**C**) inclusion complex/DOX at 12 h incubations respectively, inclusion complex/DOX group presents red fluorescence. (**D**) Cell viability inhibition analysis of inclusion complex/Nur77ΔDBD-DOX incubated with HepG2/MDR1-Bcl2 cells compared to PBS, inclusion complex alone, DOX, inclusion complex/Nur77ΔDBD, and inclusion complex/DOX. * *p* < 0.05 was considered as statistical significance.

As shown in Figure 4A-B, the cytotoxicity of the polymer materials was measured. Compared to PEI-25K, the inclusion complex shows lower cytotoxicity to the HEK293T cell and HepG2/MDR1-Bcl2 cell as the concentration increased, which indicates that the inclusion complex has good biocompatibility to reduce toxic side effects. Confocal microscopy analysis was performed to evaluate the uptake ability of DOX-loaded inclusion complex in HepG2/MDR1-Bcl2 cells. As shown in Figure 5A–C, a polymeric inclusion complex can effectively load DOX and has a better cellular uptake performance in MDR cells than free DOX, which can greatly enhance the cytotoxic effect and reduce the loss of DOX and improve the solubility of DOX. This also proves that the cyclodextrin-based polymeric complex can be an effective carrier for drug delivery. To verify the superiority of the polymeric inclusion complex in reverse MDR effect, HepG2/MDR1-Bcl2 cell viability under different treatments was measured, which is shown in Figure 5D. The result illustrates that the tumor cell viability under inclusion complex/Nur77ΔDBD-DOX treatment is better than that under complex/DOX and complex/Nur77ΔDBD treatment, which demonstrates that the inclusion complex can effectively deliver DOX and the Nur77ΔDBD gene simultaneously, thereby overcoming MDR of tumor cells and improving the efficacy of chemotherapy to achieve synergistic treatment of the tumor. The same result is displayed in Figure S5, the combination index (CI) of DOX, and Nur77ΔDBD in the inclusion complex/Nurr77ΔDBD-DOX was determined to be <0.2 with the fraction affected (*f_a_*) lower than 1 via the Chou-Talalay’s isobolographic method, which shows significant synergism of DOX and Nur77ΔDBD after integration into complex/Nur77ΔDBD-DOX.

## 4. Conclusions

The self-assembled inclusion complexes based on the two polymers (β-CD-PCL-AD and β-CD-PCL-PDMAEMA) showed better gene transfection efficacy than the gold standard PEI, showing the potential as a gene delivery system. In addition, the host-guest structures formed by the terminal AD of β-CD-PCL-AD and CD of β-CD-PCL-PDMAEMA were easy to form natural drug loading cavities, and the drug loading capacity and release capacity were superior to that of β-CD-PCL-PDMAEMA alone. In addition, we verified that DOX resistance was mainly caused by the overexpression of Bcl-2 and MDR1 genes, and we constructed a HepG2/MDR1-Bcl2 drug-resistant cell line. The polymeric inclusion complex/Nur77ΔDBD-DOX system was demonstrated to represent the superior transfection efficiency, higher cell uptake rate, and cell viability inhibition to overcome the pump efflux and non-pump drug resistance.

## Data Availability

The data is available from the corresponding author.

## Supplementary Materials

The following are available online at https://www.mdpi.com/article/10.3390/polym13081254/s1. Figure S1: ^1^H NMR spectra of β-CD-PCL-PDMAEMA, CDCl3 as solvent, Figure S2: Enrichment analysis of doxorubicin-resistant hepatoma cells in GSE125180, Figure S3: MDR1 and Bcl-2 are overexpressed in several cancer types, Figure S4: The Luciferase expression efficiency of PEI-25k only, β-CD-PCL-PDMAEMA/Nur77△DBD, inclusion complex/Nur77△DBD complexes (gradient weight ratios, from 0 to 10) incubated with HepG2 cells, Figure S5: Combination index (CI) of DOX and Nur77△DBD in inclusion complex/Nurr77△DBD-DOX against HepG2/MDR1-Bcl2 cells as a function of fraction affected (fa) determined via Chou-Talalay's isobolographic method and software CompuSyn.
