# Supplementary material for: Cyclodextrin-Based Hybrid Polymeric Complex to Overcome Dual Drug Resistance Mechanisms for Cancer Therapy
Source: Polymers (Basel). 2021 Apr 13;13(8):1254. doi: 10.3390/polym13081254 (PMC8069732; doi:10.3390/polym13081254)
Supplement: Supplementary file 1 [file polymers-13-01254-s001.pdf]

Supplementary information

Cyclodextrin-based hybrid polymeric complex to overcome du-al drug resistance mechanisms for cancer therapy

Lingjie Ke [1,†], Zhiguo Li [1,†], Xiaoshan Fan [2], Xian Jun Loh[3], Hongwei Cheng [1,*], Yun-long Wu [1,*] and Zibiao Li[3,*]

[1] School of pharmaceutical science, Xiamen University, Xiamen 361102, China; 32320191153429@stu.xmu.edu.cn (L.J.K.), lizhiguo@stu.xmu.edu.cn (Z.G.L), hongwei1026c@163.com (H.W.C)

[2] State Key Laboratory for Modification of Chemical Fibers and Polymer Materials, Donghua University, Shanghai 201620, China; xsfan@dhu.edu.cn.

[3] Institute of Materials Research and Engineering (IMRE), Agency for Science, Technology and Research (A*STAR), 2 Fusionopolis Way, #08-03 Innovis, Singapore 138634, Singapore

[*]Correspondence: hongwei1026c@163.com (H.W.C); lizb@imre.a-star.edu.sg (Z.L.); Tel.: +65-6319-4767 (Z.L.)

Supporting Figures

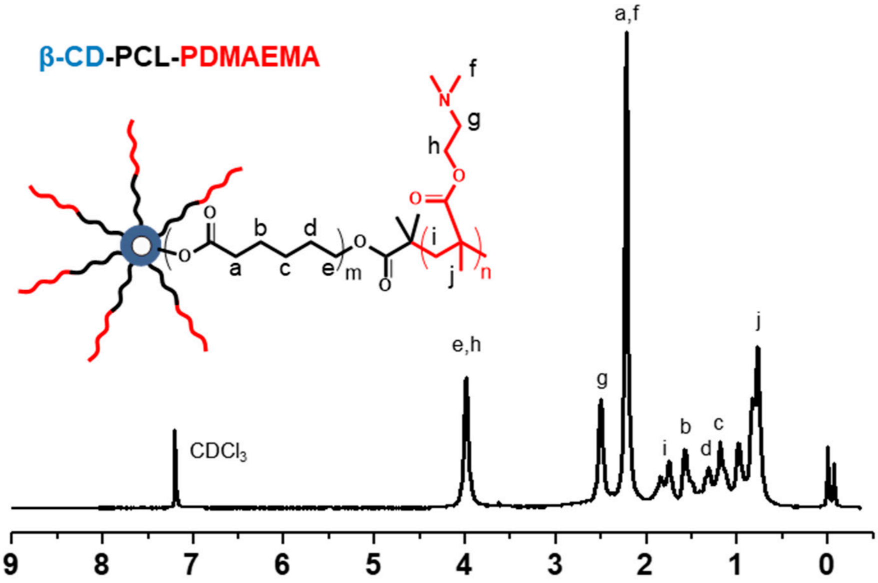

Figure S1. ^{1}H NMR spectra of β-CD-PCL-PDMAEMA, $CDCl_3$ as solvent.

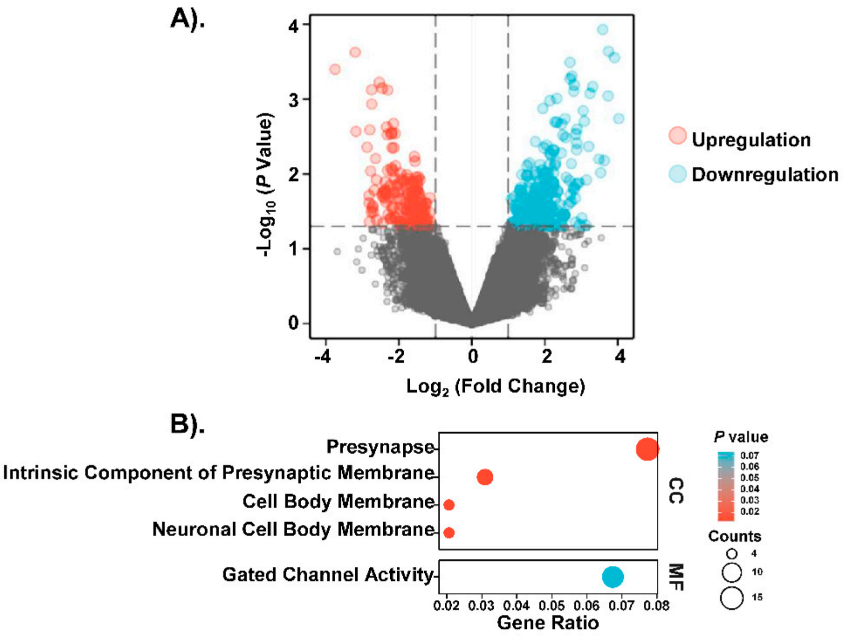

Figure S2. Enrichment analysis of doxorubicin-resistant hepatoma cells in GSE125180.

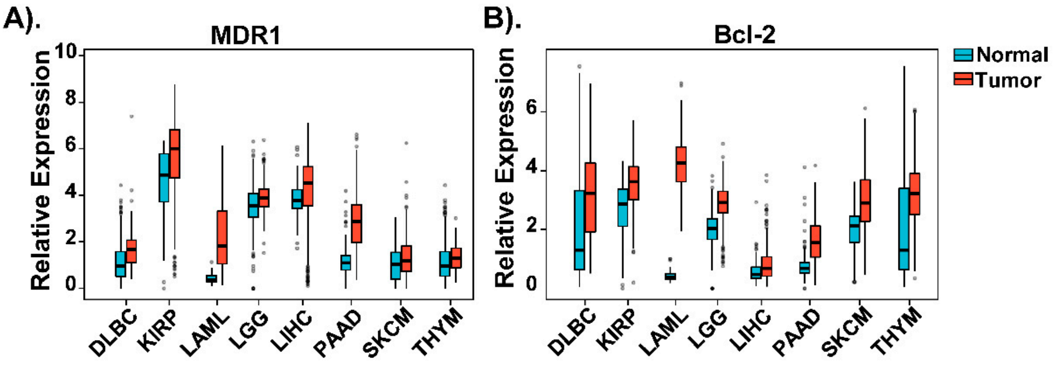

Figure S3. MDR1 and Bcl-2 are overexpressed in several cancer types.

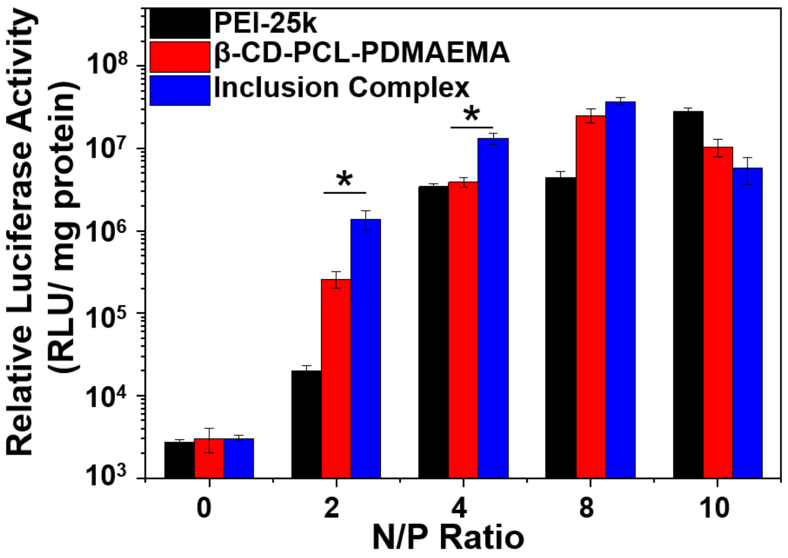

Figure S4. The Luciferase expression efficiency of PEI-25k only, β-CD-PCL-PDMAEMA/Nur77△DBD, inclusion complex/Nur77△DBD complexes (gradient weight ratios, from 0 to 10) incubated with HepG2 cells.

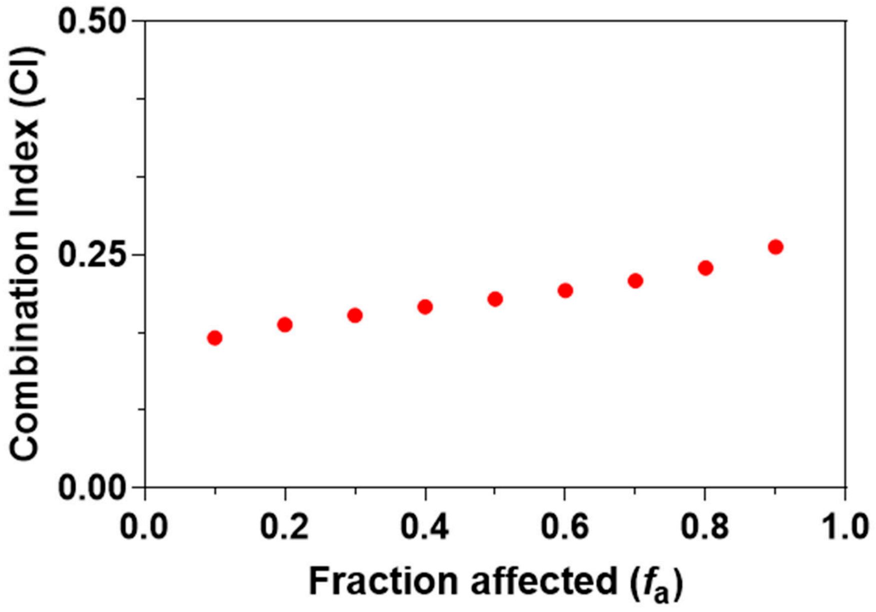

Figure S5. Combination index (CI) of DOX and Nur77△DBD in inclusion complex/Nurr77△DBD-DOX against HepG2/MDR1-Bcl2 cells as a function of fraction affected (fa) determined via Chou-Talalay's isobolographic method and software CompuSyn.
